# Community efforts to monitor and manage *Aedes *mosquitoes (Diptera: Culicidae) with ovitraps and litter reduction in east Tennessee

**DOI:** 10.1186/s12889-022-14792-4

**Published:** 2022-12-19

**Authors:** C. A. Day, R. T. Trout Fryxell

**Affiliations:** Department of Entomology and Plant Pathology, University of Tennessee, Knoxville, TN USA

**Keywords:** Community engagement, Citizen science, Passive surveillance, Source reduction, Container mosquitoes, *Aedes albopictus*, *Aedes triseriatus*, *Ochlerotatus*

## Abstract

**Background:**

East Tennessee (USA) is burdened by mosquito-borne La Crosse virus disease, but minimal resources for mosquito surveillance, management, or related community education exist in the region. To address these needs, we developed a program to train middle and high school educators in basic medical entomology. The educators then used their skills in the classroom to teach students about La Crosse virus disease and conduct mosquito collection experiments. As a case study of a potential application of classroom-collected data, we also partnered with a local non-profit organization to assess the potential for a volunteer litter cleanup to reduce mosquito populations in a Tennessee neighborhood.

**Methods:**

Our first objective was to investigate the ability for educators and their students (schools) to collect high-quality mosquito surveillance data. In 2019 and 2020, we collected *Aedes* (Diptera: Culicidae) eggs during the same study period as schools and assessed whether data collected by schools reflected the same findings as our own data. Our second objective was to investigate the impact of a volunteer litter cleanup event on *Aedes* mosquito abundance. In 2021, we collected *Aedes* eggs before and after a neighborhood trash cleanup while schools conducted their own mosquito egg collections. Using the school collections as non-treatment sites, we used a Before-After-Control-Impact analysis to determine if there was a significant decline in egg abundance after the cleanup.

**Results:**

In 2019, mosquito abundance trends were similar between our data and school data but differed significantly during some weeks. After refining our protocols in 2020, school data was highly similar to our data, indicating that schools consistently collected high-quality surveillance data in the program’s second year. In 2021, we found a significant decline in *Aedes* egg abundance after the litter cleanup event in comparison to the schools, but the number of adults reared from those eggs did not differ between sites after the cleanup.

**Conclusion:**

The results of our work demonstrate the potential for community-driven programs to monitor mosquito abundance trends and for volunteer-based cleanup events to reduce the burden of *Aedes* mosquitoes. In the absence of infrastructure and resources, academic-community partnerships like the ones evaluated here, provide opportunities to help resource limited areas.

## Background

Emerging and re-emerging vector-borne diseases (VBDs) are growing threats to human health, but most of the United States (US) lacks programs for vector surveillance and control, leaving communities ill-prepared to detect and respond to VBD outbreaks [1–3]. This is due in-part to the resource-intensive nature of vector surveillance, which requires trained entomologists, acquisition of trapping supplies, laboratory space for arthropod rearing and identification, and contamination-free bio-secure space for pathogen detection [3]. A lack of consistent surveillance leaves communities at risk for unpredicted VBD outbreaks and hinders our ability to understand the long-term effects of climate change on vector populations and disease transmission [4]. In the absence of robust publicly funded programs for vector surveillance and control, there is growing support for the implementation of community-based solutions (e.g., community engagement or citizen science) to amplify data collection efforts and foster partnerships between public health agencies and community stakeholders [5].

Community-driven science is gaining recognition for its potential to supplement professional surveillance and control of mosquito populations [6]. Participants in community-based programs are tasked with applying simple methods for vector surveillance and control, such as larval habitat reduction [7], manual capture of adult mosquitoes [8], or the deployment of passive traps that collect mosquito eggs or gravid adults [9–11]. Such efforts have provided valuable supplementation of professional surveillance and control by achieving significant reductions in mosquito populations and for detecting invasive species [10–13]. There are significant limitations to community-collected data, but those limitations are often outweighed by the benefits of upscaling the geographic range of collections, especially when combined with professional mosquito surveillance [11, 13]. Critically, in areas where VBDs are persistent but prevention efforts are insufficient, community-based programs may be the most realistic method for protecting communities [5]. Such a need exists in the southern Appalachian region of the US, including east Tennessee, where resources for VBD prevention are minimal and mosquito-borne La Crosse virus (LACV) causes neuroinvasive disease in children annually [14].

La Crosse virus causes more pediatric neuroinvasive disease than any other arbovirus in the US [14]. Neuroinvasive LACV disease can create a substantial economic burden for the families and communities of infected individuals, especially in severe cases that lead to lifelong cognitive disorders [15–17]. The virus is transmitted by mosquitoes (Diptera: Culicidae) in the genus *Aedes* that use water-holding containers (e.g. discarded tires) as habitats for immature development [18, 19]. The principal LACV vector is *Aedes triseriatus* (Say, 1823) [20, 21], while two invasive species, *Ae. albopictus* (Skuse, 1895) and *Ae. japonicus japonicus* (Theobald, 1901), act as secondary vectors [22–24]. Most cases of LACV disease occur in the Appalachian region [25, 26], and risk factors include high abundances of *Aedes* mosquitoes, time spent outdoors, and artificial container density near the household [27, 28]. Although these risk factors can be reduced by behavior changes, community awareness of LACV disease and its risk factors are low, and there are many knowledge gaps in risk prediction that make it challenging for public health officials to prevent the disease through targeted intervention or education [16, 17, 29]. The greatest hindrance to LACV disease prevention in east Tennessee is that most counties with high disease risks have no public VBD surveillance or control resources, leaving children unprotected from LACV infections. As such, there is a continued need for programs that increase community awareness and provide surveillance of LACV and its vectors. In the absence of policy-based support for LACV disease prevention, community-based programs could provide much-needed surveillance data and community awareness.

In 2019, we initiated a community health and science-based engagement program [37] to enhance LACV surveillance and the community awareness of LACV disease in east Tennessee, where LACV infections have been reported annually since 1997 [14, 18, 26]. Our program, called MEGA:BITESS (Medical Entomology and Geospatial Analysis: Bringing Innovation to Teacher Education and Surveillance Studies), trains science educators from middle and high schools in east Tennessee in the field of medical entomology. After receiving training, educators use the knowledge gained to develop and implement lesson plans that raise awareness of mosquito biology and LACV disease risk. To promote high-impact learning, the educators also organize student-led mosquito collection experiments. The primary goal of MEGA:BITESS is to raise community awareness of locally important mosquito-borne diseases, but the data collected during student-led mosquito collections may also be useful for understanding LACV vector ecology and abundance trends. Presuming these data are of high quality, long-term collections by educators and students could provide insights into how year-long weather patterns impact mosquito abundance and species compositions, how climate change is impacting mosquito populations, or how mosquito abundance relates to annual LACV disease incidence. However, there are many challenges associated with community-engaged science, and data derived from community-driven projects are not always useful for hypothesis-driven research [30].

Here we present the results of the first three years of our community engagement program, MEGA:BITESS. Our overall goal was to develop a partnership with community members to determine the feasibility and efficacy of a community-driven mosquito surveillance program, and within the first three years of the program we pursued two objectives related to that goal. Objective 1 was to determine if mosquito collections by participants in our community engagement program (hereon referred to simply as schools) accurately assess *Aedes* mosquito abundance trends. We tested the hypothesis that mosquito abundance trends will be similar in data collected by schools and by entomologists at the University of Tennessee – Knoxville (UTK). Objective 2 was to conduct a case study to demonstrate the usefulness of a community-driven surveillance program as a supplement to our own research activities. Specifically, we investigated the impact of a neighborhood litter cleanup organized by a local non-profit organization on the abundance of *Aedes* mosquitoes using data collected by schools as a control dataset to test the hypothesis that a neighborhood litter cleanup would reduce the abundance of *Aedes* mosquitoes in the area. It is already known that high densities of human-made containers (e.g., buckets, cups, litter, bird baths, and used tires) lead to elevated mosquito populations [28, 31] and are associated with elevated risk for LACV infection [32]. An effective method for reducing the burden of container mosquitoes is reduction of potential larval habitats, accomplished via container removal either by professionally trained crews (e.g., sanitation, public health personnel, entomologists) [33–36]. Although source reduction, also referred to as “tip and toss”, is a well-known method for mosquito population control, the effects of volunteer-based litter cleanups on *Aedes* mosquito populations have not been measured.

## Materials and methods

### Study period

This study was conducted from 2019 to 2021, the first three years of our community engagement program. The first objective of the study was completed in the first two years (2019–2020) and the second objective was completed in the third year (2021). Five-day educator training workshops were held in June each year. In 2019, mosquito collection was conducted from early August to early October (calendar weeks 31–40), followed by mosquito rearing and identification from December 2019 – February 2021. In 2020, the mosquito collection period ran from late August to early October (calendar weeks 35–40), followed by mosquito rearing and identification from November 2020 – March 2021. In 2021, the mosquito collection, rearing, and identification took place over the same timespan as the previous year.

### Study participants

Participants in our community engagement program include educators and their students (schools) in east Tennessee. In 2019, 17 educators from 13 schools in six different counties participated. In 2020, 15 of those educators from 12 schools in six counties returned to participate in the program’s second year [37]. In 2021, 13 educators (nine returning and four new) from 12 schools in seven counties participated in the program. Hereon, school is used to refer to individual educators and their students (e.g., in 2019, 17 schools participated). UTK mirrored the methods used by schools in each study year to create comparison datasets. Objective 2 included the participation of a local non-profit organization, Keep Knoxville Beautiful (KKB) (https://www.keepknoxvillebeautiful.org/) during KKB’s inaugural east Knoxville neighborhood cleanup event. The study has been performed in accordance with the University of Tennessee and approved by the Institutional Review Board (UTK IRB-19-05046-XP).

### Mosquito Collection

The mosquito collection protocols used in this study were described in detail by Trout Fryxell et al. [37] and are described briefly here. The same collection protocol was used in each year and objective of this study. Mosquito eggs were collected using ovitraps, which are 750 mL black plastic cups lined with seed germination paper (10.2 cm in width; SD3815L, Anchor Paper, Plymouth, MN, USA; hereon referred to as egg papers) and filled with 500 mL of an infusion of bovine liver powder (Fisher Scientific, Waltham, MA, USA) and water. The liver powder infusion was made by adding ½ teaspoon of bovine liver powder (#02900396 MP Biomedicals, Solon, OH, USA) to 2.5 gallons (9.46 L) of dechlorinted water. The abundance of eggs oviposited on the seed germination papers are commonly used to assess container mosquito abundance [38–42]. Ovitraps were deployed for 7-day collection periods. Each week, the previous liver-powder infusion was discarded and a new liver powder infusion was added to the ovitraps along; concurrently, seed germination papers were replaced. The collection week was considered to be the week that the seed germination papers were collected and removed from the field.

### Egg holding

In 2019, egg papers were dried in the open air before being stored in paper envelopes. Schools stored egg papers in their classrooms, and UTK stored egg papers in a mosquito rearing laboratory. The UTK mosquito rearing laboratory was held at an ambient air temperature of approximately 26**°**C, but air temperature was not specified in school classrooms. In 2020, an identical egg holding protocol was employed during the first study week, but due to low hatch rates in 2019 and visible desiccation of eggs collected during the first study week of 2020, the egg holding protocol was adjusted. Beginning in the second study week of 2020, UTK began to hold egg papers in 18 oz plastic Whirl-Pak bags (Whirl-Pak, USA) and were kept in insulated coolers containing a plastic oviposition cup holding approximately 300 mL of water to maintain increased humidity around the eggs. Schools were informed of this change in the protocol via email but were not provided with WhirlPak bags. Seven of the 15 schools adapted their egg holding protocols by holding the plastic envelopes in sealed plastic containers of varying sizes, sometimes with a water source like moist cotton balls for added humidity. The other schools continued with the 2019 egg holding protocol throughout the 2020 collection period. Because storing eggs in plastic significantly increased the success of rearing mosquitoes from eggs to adulthood [37], all study participants used plastic WhirlPak bags for egg paper storage in 2021.

### Mosquito rearing and identification

Each year after the study periods were completed, we collected egg papers from schools and counted the number of hatched (noted by an open egg cap) and not hatched (e.g., embryonating, turgid) eggs collected by schools and UTK using a dissection microscope. We reared mosquitoes from egg-to-adulthood in an environmentally-controlled BSL-2 rearing facility at approximately 26 °C and a day-night cycle of 16 h light : 8 h darkness [43]. To hatch the eggs, we submerged the egg papers in mosquito breeding chambers (BioQuip, Rancho Dominguez, CA, USA) containing 500 mL of a liver-powder and yeast infusion; the infusion is as described above, but with an additional 1.5 g of active dry yeast (AB Mauri, St. Louis, MO, USA). In 2019, we submerged the eggs for three periods of 24 h separated by 24-hours of drying in the open air. In 2020 and 2021, we submerged the eggs for two periods of 24 h separated by 24 h of drying in the open air. We grouped the eggs by week of collection and hatched each group on a weekly basis to preserve shelf space in the rearing facility. We killed adult mosquitoes in the freezer and identified them to sex and species using microscopic characters [44].

### Objective 1: Assessment of community mosquito collections (2019–2020)

 Schools and UTK used similar mosquito collection protocols during the same collection periods in 2019 and 2020 to create comparable datasets. In 2019, schools and UTK collected mosquito eggs for 10 consecutive weeks (calendar weeks 31–40). Schools deployed 10 ovitraps around their campuses while UTK deployed one ovitrap at 26 sites in Knox County, TN. In response to feedback from educators about their workload in 2019, the study period and number of traps deployed by schools was reduced in 2020 (the second year of the program). Additionally, UTK increased the number of traps placed per site to better reflect the number of traps placed by the schools. In 2020, both schools and UTK deployed ovitraps for six consecutive weeks (calendar weeks 35–40). Schools deployed six ovitraps on their campuses while UTK deployed 3 ovitraps at 31 sites in Knox County, TN.

Schools deployed ovitraps on school campuses, which were located in multiple east Tennessee counties, while UTK deployed ovitraps throughout Knox County, TN. To reduce geographical and site-level biases in the datasets, UTK deployed ovitraps in a diverse array of sites in Knox County, including parks, cemeteries, and homes located in urban, suburban, and industrial zones. Most of the UTK sites were previously used for mosquito surveillance by Rowe et al. [45] and were known to include a mix of high and low seasonal *Aedes* spp. abundances. At the end of each study period, educators returned their egg papers to us. We counted the eggs collected in each trap using a dissecting microscope.

Because schools tested their own classroom-driven hypotheses, they sometimes placed their ovitraps in areas that were unlikely to attract mosquitoes (e.g., some ovitraps were deployed in unshaded parking lots to test the hypothesis that mosquitoes prefer to lay eggs in forested areas). To avoid biases resulting from the school-specific study designs, only the three ovitraps at each school that collected the most eggs each week were considered in statistical analyses and visualizations. Summary statistics were calculated with R version 4.0.5 [46] and included the total number of eggs collected, mean eggs collected with standard deviation, total number of mosquitoes reared to adulthood, total number of mosquitoes reared to adulthood per species, and the mean percentage of embryonating eggs reared to adulthood. Boxplots were used to visualize temporal trends in egg abundance and subsequent abundance of adults reared from egg collections. The boxplots were created with the R package ggplot2 version 3.3.5 [47] using the package’s default definition of outliers (1.5 times greater than the median) and without visualizing the outliers to maintain reasonable y-axes.

Generalized linear mixed effect models (GLMMs) were used to formally test the hypothesis that weekly egg abundance was not significantly different when data were collected by schools or UTK. The GLMMs were fitted using the R package glmmTMB (version 1.1.2.3) [48] using a negative binomial distribution with a log-link function to account for overdispersion in the models. Two models were fitted for each study year (2019 and 2020): one with number of eggs as the outcome, and another with the number of mosquitoes reared to adulthood as the outcome. Fixed effects included calendar week of mosquito collection, data collector (schools or UTK), and the interaction of calendar week and data collector. Trap location was included as a random slope to account for repeated sampling. A significant interaction effect implied that egg abundance trend differed significantly between data collected by schools and UTK [49]. Statistical significance of interaction effects were tested by comparing the full models to models that lacked the interaction term using likelihood ratio tests. To better understand which calendar weeks were driving overall significant differences in trends, p-values from each level of the interaction were analyzed using calendar week 37 as the reference level.

### Objective 2: Case study- *Aedes* management via volunteer litter cleanup (2021)

 On 18 September 2021, the local non-profit organization KKB organized a volunteer-based litter cleanup in a Knoxville, TN neighborhood. KKB divided the neighborhood into 11 subsections and dispersed 18 drop-off locations throughout the subsections. 146 volunteers collected litter for approximately four hours throughout the subsections. Volunteers were not trained in medical entomology or the role litter has with mosquito production; instead, volunteers were instructed for personal safety and instructed to stay in public spaces, avoid private yards, and to focus on litter that was accessible to pick up and discard. The litter removed included items that could not hold water (e.g., clothing, cardboard, food wrappers, Styrofoam, and glass) and items that could hold water. Those items included plastic containers (e.g., five-gallon buckets, take-out boxes, cups, etc.) and used tires that likely served as water-holding habitat for *Aedes* mosquito larvae.

UTK placed nine ovitraps throughout the neighborhood receiving the litter cleanup (KKB sites). Two weeks of collection were completed prior to the trash cleanup (calendar weeks 36 and 37), followed by three collections after the cleanup (calendar weeks 38–40). These collections aligned with the 2021 school collections, which occurred at non-treatment sites from calendar weeks 35 to 40. Only the data from the six participating schools located in Knox County, TN were used for comparison with KKB sites to avoid excessive geographic biases from schools outside of the county. Schools deployed 6 ovitraps for the collection period, following the same procedures as in 2020 (described in Objective 1). We excluded two Knox County schools from the analysis because they collected an extremely small number of eggs, leaving four total schools. As in Objective 1, at schools, only the 3 ovitraps that collected the most eggs each week at were used for summary statistics and analyses.

Summary statistics were calculated using R version 4.0.3 [46] including total eggs collected, mean eggs per collection with standard deviation, total number of each species reared to adulthood, the mean number of mosquitoes reared to adulthood with standard deviation, and the mean percentage of embryonating eggs reared to adulthood. Boxplots were created with ggplot2 version 3.3.5 [47] using the same parameters described in objective 1 to visually assess changes in egg abundance before and after the cleanup. To test the hypothesis that a neighborhood cleanup reduced mosquito abundance, another GLMM was used. In this case, the analysis was equivalent to a Before-After-Control-Impact (BACI) analysis [50]. The GLMMs were fit using R package glmmTMB (version 1.1.2.3) [48]. Schools represented untreated controls (i.e., where no litter cleanup occurred), and KKB sites represented treatment sites (i.e., where the litter cleanup occurred). The full model was fit using a negative binomial distribution with a log link function, using the number of *Aedes* eggs as the outcome variable. Based on the results of objective 1, adults were not used as an outcome. Fixed effects included treatment or control (KKB sites or school sites), pre-treatment or post-treatment, and an interaction between those two effects. A significant interaction term implied that the number of eggs before and after the litter cleanup was significantly different between KKB sites (where the litter cleanup occurred) and the schools (where no cleanup occurred). Trap location was included as random intercepts to account for repeated sampling. The significance of the interaction was tested by comparing the full model to a model lacking the interaction effect with a likelihood ratio test.

## Results

### Objective 1: Assessment of community mosquito collections

**Fig. 1:**
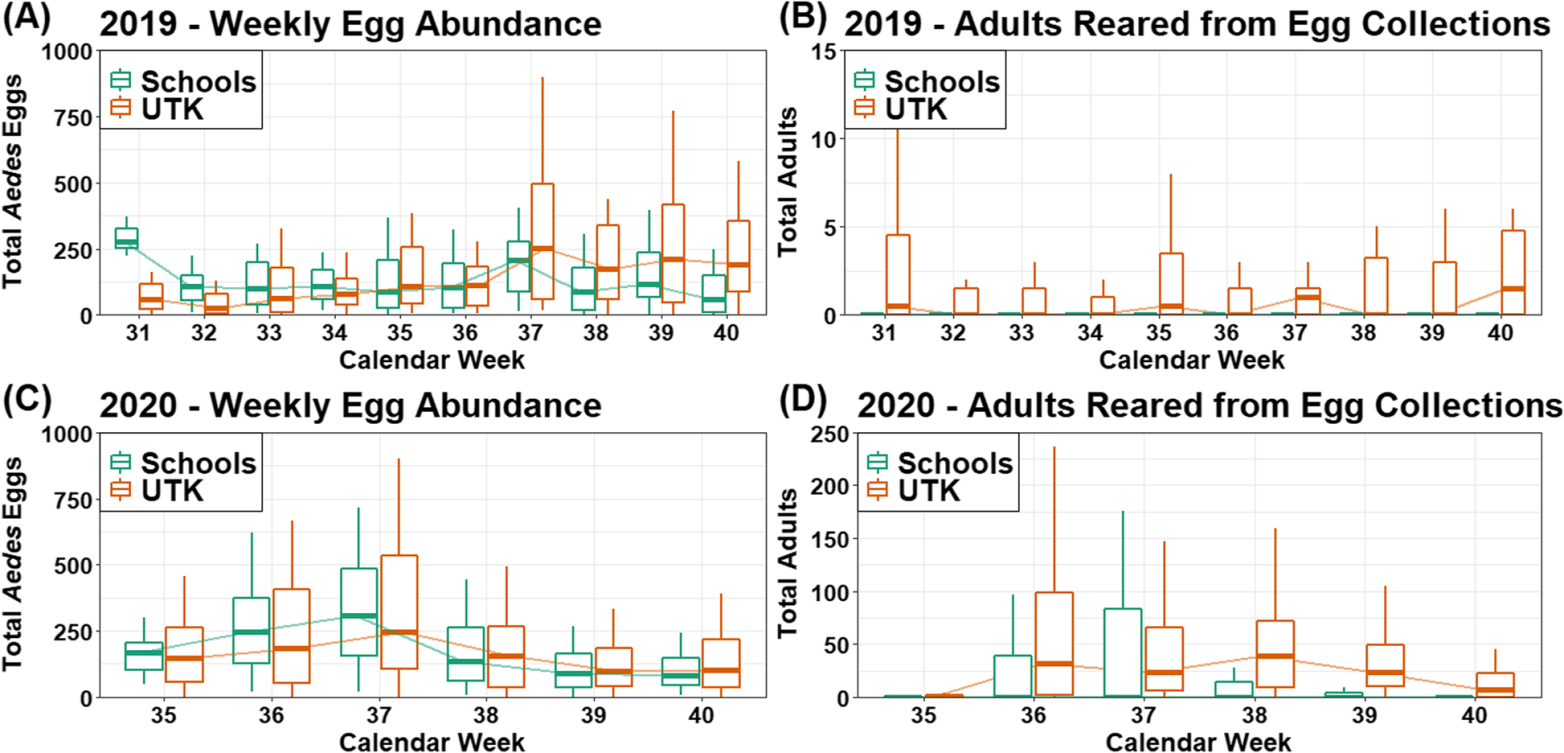
Weekly number of eggs and adults reared from each egg collection in 2019 and 2020 by schools and UTK

In 2019, schools collected nearly two times more eggs than UTK in total, but UTK collected approximately 112 more eggs per trap on average (Table 1). Although schools collected more eggs overall, more eggs were reared to adulthood from the UTK collections. *Aedes albopictus* was the only species to emerge from the eggs collected by both schools and UTK. Rates of egg-to-adulthood were poor for UTK and school collections, but a higher proportion of embryonating eggs were successfully reared to adulthood from UTK collections. Egg abundance trends were visually similar between UTK and schools for most of the study period (Fig. 1). The week with the lowest median egg abundance differed between schools (calendar week 40) and UTK (calendar week 32), but the maximum median egg abundance was calendar week 37 for both UTK and schools. Overall, the trends were significantly different as indicated by the significant interaction between calendar week and data collector (i.e., Schools vs. UTK) in the GLMM (χ^2^(9) = 35.01, p < 0.001), due primarily to the final study week (calendar week 40) when schools had a substantial decline in egg abundance while UTK abundance remained similar to the previous week. Few eggs were successfully reared to adulthood each calendar week (Fig. 1), and the trends in abundance based on the number of adults reared from each egg collection were significantly different between UTK and schools (χ^2^(9) = 45.78, p < 0.001). Based on a visual comparison, there was no correlation between the weekly number of eggs collected or the weekly number of adults reared from egg collections (Fig. 1).

**Table 1:** Descriptive analysis of mosquito egg collections from schools and UTK in 2019 and 2020. Mosquitoes were reared from egg to adult for species identification

Year	Site Type	Number of Collections	Total Eggs	Mean Eggs per Trap (Std Dev.^1^)	Total Emerged Adults		Total *Aedes* *albopictus* adults	Total *Aedes* *triseriatus* adults	Total *Aedes japonicus* adults	Mean Egg-to-Adult Percentage (Std Dev.^1^)
**2019**	Schools	1317	95,256	72.33 (113.7)	1120	1,099		0	0	2.8% (22.9%)
	UTK	269	49,324	184 (219.2)	3122	3,122		0	0	9.8% (41.8%)
**2020**	Schools	566	71,903	127 (161.6)	9,441	7,345		475	0	12.1% (52.7%)
	UTK	527	111,185	210.2 (217)	29,960	20,737		9,177	24	31.3% (43.8%)

^1^Standard deviation

In 2020, UTK collected more eggs in total and on average than schools (Table 1). Egg-to-adulthood rates were substantially higher than in 2019 for UTK and schools, but were still very low for schools. More than three times as many mosquitoes survived to adulthood from UTK collections than from school collections, and the average rate of survival from egg-to-adulthood was approximately two times greater among eggs collected by UTK. Three species emerged from eggs collected by UTK (*Ae. albopictus*, *Ae. triseriatus*, and *Ae. japonicus*), and two species emerged from eggs collected by schools (*Ae. albopictus* and *Ae. triseriatus*), but *Ae. albopictus* was the most abundant species regardless of data collector (Table 1). Egg abundance and weekly abundance trends were visually similar between UTK and schools (Fig. 1), with a steady rise until calendar week 37 followed by a steep decline through calendar week 40. Statistically, the trends were not significantly different (χ^2^(5) = 1.83, p = 0.87). As in 2019, the weekly trends in adults reared from egg collections were significantly different between UTK and schools (χ^2^(5) = 29.11, p < 0.001) and did not reflect weekly egg abundance trends.

### Objective 2: Community mosquito management via neighborhood litter cleanup

**Fig. 2:**
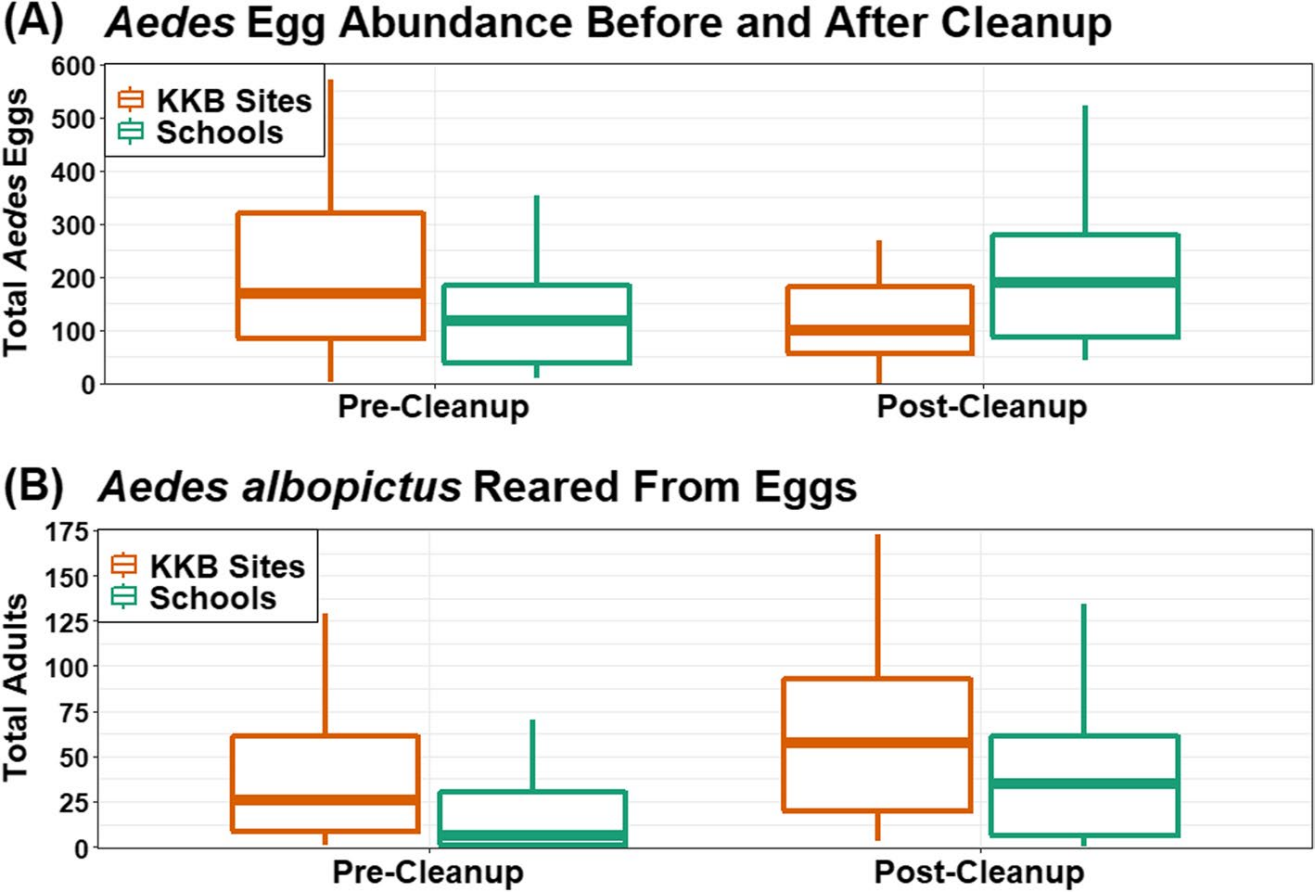
Weekly number of eggs collected at Keep Knoxville Beautiful (KKB) sites and Schools before and after the litter cleanup event

*Aedes albopictus* and *Ae. triseriatus* were collected at KKB sites and schools, and *Ae. japonicus* was collected at schools but not at KKB sites (Table 2). At all sites, *Ae. albopictus* was the most common species. Egg-to-adulthood rates were substantially higher for schools in 2021 than in 2019 or 2020 (objective 1), but the 2021 analysis only included a subset of the schools (Table 2). At the KKB sites (i.e., treatment sites), average egg abundance declined from 201.1 eggs per ovitraps pre-cleanup to 135.0 eggs post-cleanup (Table 2). Concurrently, at schools (i.e., control sites), egg abundance increased from 109.6 eggs per ovitrap prior to cleanup to 198.7 eggs per ovitrap post-cleanup. Results of the BACI analysis indicated that the decline in egg abundance at KKB sites after the litter cleanup was statistically significant (χ^2^(1) = 6.87, p = 0.009). Figure 2 shows that weekly egg abundance was greater at KKB sites than at schools prior to the cleanup, but that schools had greater egg abundance than KKB sites after the litter cleanup event. Those egg abundance trends were not reflected in the adult abundance trends, as evidenced in Fig. 2. Although the majority of eggs were *Ae. albopictus*, the number of *Ae. albopictus* eggs reared to adulthood increased after the trash cleanup for schools and UTK, reflecting higher hatch rates post-cleanup instead of decreased egg abundance.

**Table 2:** Descriptive assessment of the litter cleanup event on *Aedes* egg abundance. Mosquitoes were reared from egg collections to adulthood for species identification

Collection Period	Collection Site	Number of Collections	Total Eggs	Mean Eggs (Std. Dev.^1^)	Total *Aedes albopictus*	Total *Aedes triseriatus*	Total *Aedes japonicus*	Mean Total Adults (Std Dev.^1^)	Mean Egg-to-Adult Percentage (Std Dev.^1^)
**Pre-Cleanup**	KKB Sites (Treatment)	18	3,620	201.1 (158.7)	890	41	0	66.50 (73.44)	29.2% (41.7)
	Schools (Control)	24	3,939	109.6 (109.2)	685	328	0	42.21 (59.10)	22.6% (19.1)
**Post-Cleanup**	KKB Sites (Treatment)	25	3,376	135 (112.4)	1,258	27	0	64.25 (48.73)	52.6% (66.4)
	Schools (Control)	37	7,353	155.6 (132.6)	1,531	386	13	52.16 (51.22)	26.2% (19.5)

^1^Standard deviation

## Discussion

We aimed to collaborate with educators in east Tennessee to develop a program that would simultaneously increase community awareness of the locally important LACV disease, foster high-impact learning and enthusiasm for students in STEM courses, and produce mosquito surveillance data that would be useful for hypothesis-driven inquiries [37]. In this study, we found that educators and their students in our program can collect *Aedes* egg abundance data that closely reflects data collected by our entomology laboratory. This result was inconsistent in the first year of the program, but in year two (2020), it was clear that schools collected high-quality mosquito surveillance data. If schools were our only source of egg abundance data during our 2020 study period, we would have reached the same conclusions about abundance trends as if we collected the data ourselves. With confidence that our community participants can collect high-quality data, we conducted a case study to amplify the work of those students to demonstrate the potential utility of their independent studies, and in doing so found that *Aedes* egg abundance significantly declined in a Knoxville, TN neighborhood after a volunteer-led litter cleanup. Though not without limitations, this study represents the potential for community-engaged science to provide a needed supplement to mosquito surveillance in east Tennessee.

### Objective 1: Assessment of community mosquito collections

 In 2019, the process of training educators to use our egg collection protocol presented several challenges. Most notably, participants informed us that the quantity of traps and length of the collection period was overwhelming, and we recognized that our egg holding protocol was causing high rates of egg desiccation. In 2020, we reduced the number of traps and the study period and altered the egg collection protocol, and the results of the community-based mosquito collections improved. It is unclear if the improved similarity in egg abundance trends that we saw from 2019 to 2020 was due to the additional year of hands-on egg collection experience that schools gained in 2019 or a result of year-to-year refinement of our program, but it was evident that our improved egg holding protocol resulted in better survival of the collected mosquitoes. Ongoing retention of participants who have agreed to volunteer as peer mentors to new participants will hopefully assist in ensuring that future participants are successful in their initial attempts at mosquito collections [37].

Based on our findings, egg abundance trends of container-inhabiting *Aedes* spp. seem to correlate at a broad scale, even though significant differences in raw abundance exist at fine scales [45, 51]. This is a critical distinction: two sites may have vastly different total mosquito abundance, but climate-driven temporal abundance trends could still be the same between the sites. Notably, assessments of similar community-driven mosquito surveillance programs have focused on mosquito presence and community composition, but not on abundance trends, which may limit potential data uses [8, 11, 13]. In the long-term, data from our program will be useful for investigating relationships between seasonal LACV vector abundance trends and climate change [4, 52], which likely plays an important role in late-summer rates of LACV transmission [29].

A notable limitation of our approach was our inability to successfully rear adults from the eggs to adequately mirror egg abundance data. This limitation limits our ability to assess species-specific population trends and needs to be addressed in future years. Our goal was to create a reasonable egg holding protocol that the educators could use with limited supplies and time, while allowing for egg delivery from classrooms to our research laboratory. The egg holding method in year one (2019) was highly convenient, but insufficient for protecting the eggs. The new method is better for protecting the eggs, but requires more effort, expense, and inconvenience when storing and organizing eggs. By the third year of our program (2021), school collections successfully yielded all three container *Aedes* species that oviposit on egg papers in east Tennessee [53], two of which are invasive species (*Ae. albopictus* and *Ae. japonicus*). Even with difficulties in measuring species-specific abundance trends, the schools can likely detect the introduction of other invasive container *Aedes.* With continued engagement with the educators, improvements to egg holding in the classroom and hatching protocols in the laboratory, we can most likely achieve greater egg hatching rates from school collections in future years. However, it is also important to note that wild-caught mosquito egg hatching is highly variable and affected by a multitude of factors including egg desiccation in the field, geographic origin of the mosquitoes, predation, hatch inhibition due to the presence of con-generic species in the water, and bet-hedging behaviors [54–58]. For example, in one study, *Aedes* hatch rates ranged from 60–80% in early summer but declined to 10–20% in Autumn [34]. An approach where eggs are identified to species without hatching would likely be more accurate, but distinguishing characteristics are inconsistent between eggs of some species (e.g., *Ae. triseriatus* and *Ae. j. japonicus*) [53, 59]. Importantly, [39] compared the number of eggs in an ovitrap to biting *Ae. albopictus* with human landing rates and found a significant correlation that for every five eggs one mosquito bite occurred. While we cannot make this claim, it demonstrates the value of egg data.

We use ovitraps in our program because they are inexpensive and easy for community members to deploy appropriately. They require minimal training and do not need chemical attractants or batteries to be effective. However, upscaling our community engagement project to obtain more mosquito surveillance data would be challenging. Although egg collections using ovitraps are convenient, subsequent egg counting, mosquito rearing, and adult mosquito identification requires ample space and person-time. There are potential alternatives to the ovitrap method that maintain the advantages of simplicity and inexpensiveness such as “e-entomology”, where participants send photos of collected mosquitoes to entomologists for identification to be used in a large-scale community science project for container mosquito surveillance [11]. Similarly, the Müeckenatlas project in Germany tasks participants with manually capturing mosquitoes in jars and submitting the specimens to entomologists via mail [8]. The choice of approach should depend not only on availability of entomological resources, but also the goal of the program. Our program is a form of “train-the-trainer” model, where we train highly educated trainers (i.e., middle and high school educators), who then teach their students about entomology, LACV disease, and mosquito collections. Our approach is collaborative and highly targeted to the needs of the local community, and therefore our methods involve direct engagement with community stakeholders and requires less geographic reach than some other programs. Importantly, because LACV is transmitted from female to egg use of ovitraps will allow us to conduct LACV surveillance in the future as well.

### Objective 2: Community mosquito management via neighborhood litter cleanup

 As mentioned, schools designed their egg collections around hypotheses developed by students. One school asked a question related to litter (e.g., are there more mosquito eggs near a site with litter?), which inspired us to collaborate with the local non-profit organization Keep Knoxville Beautiful to test the effects of their litter cleanups on the mosquito abundance. Knowing that schools collected high-quality mosquito abundance data in 2020, we were confident that schools could provide a quality control dataset for comparison with data that we collected at the litter cleanup sites. We found that *Aedes* egg abundance declined significantly after the cleanup event in comparison to schools (control sites). As in objective 1, our adult emergence rates did not adequately reflect the egg abundance data, which may have been a failure of our rearing protocol or a product of some external factor (e.g., excessive egg desiccation at oviposition site prior to collection). Nevertheless, results of the egg collections are exciting because it implies that this volunteer-based litter cleanup may have reduced the abundance of *Aedes* mosquitoes.

Objective 2 was primarily meant to serve as a case study of how a community engagement program can be useful for inquiry-driven research, and our study design was not robust enough to prove the impact of litter cleanups on *Aedes* populations. Replication of our results with the addition of adult trapping methods is necessary to confidently conclude that cleanup events of this nature significantly impact mosquito populations. Theoretically, the reduction of containers should reduce the number of potential oviposition sites, which may subsequently increase the number of eggs laid in ovitraps and cause this approach to be unreliable in this context. However, immature-stage metrics (e.g., number of containers with pupae) can predict adult *Aedes* abundance [60], and in this scenario there may have been many containers with larvae and pupae that were discarded in the litter cleanup which reduced subsequent abundance of gravid *Aedes* in the short term. Most research on source reduction as a method for *Aedes albopictus* control has involved professional mosquito control programs working with homeowners to reduce household container density [61]. Homeowner education to achieve voluntary source reduction can result in significant reductions in the abundance of containers and mosquitoes [34, 62], but door-to-door source reduction campaigns are resource-intensive and do not often achieve sustainable results [34, 63].

The impact of litter cleanups on mosquito abundance likely depends on a combination of socioeconomic metrics and public resource availability. The neighborhood that received a cleanup in this study has a history of destructive urban renewal policies that reduced the area’s socioeconomic wellbeing and access to public amenities relative to the rest of the city [64, 65]. Socioeconomically-disadvantaged neighborhoods are sometimes correlated with more litter generation [66], and based on our observations as participants in the cleanup event, the neighborhood was littered with a plethora of large water-holding containers like tires, buckets, and non-degradable food containers (e.g., cereal cups). Even very small containers like bottle caps and discarded chip bags can support *Aedes* larval development, and an innumerable quantity of such containers were collected throughout the neighborhood [34, 67]. The effect of a litter cleanup would likely be diminished if most containers supporting mosquito populations are on inaccessible private properties. In this case, whether due to poor waste removal infrastructure or another concern, the neighborhood contained a plethora of litter in publicly accessible spaces that could contribute to mosquito population growth. If litter cleanups are combined with community and homeowner education, the effects of container removal may be amplified, especially in neighborhoods where most containers are on private property.

## Conclusion

After methodological improvements following the first year of a community engagement program, educators and students from middle and high school classrooms collected high-quality surveillance data that approximated data collected by a university laboratory. The value of these data was evident in a case study investigating the effects of a neighborhood litter cleanup on *Aedes* mosquito abundance, which would have been impossible given our limited resources without the participants in our community engagement program. The efforts of a local non-profit organization and dozens of volunteers reduced mosquito abundance in a Knoxville, Tennessee neighborhood. These partnerships between our university and community stakeholders represent an exciting opportunity to apply academic resources to empower communities to understand and reduce the burden of mosquitoes in the region. Better policy-based infrastructure for waste removal and mosquito surveillance would be preferable, but in its absence, these community partnerships enabled this work that both monitored and reduced the mosquito burden of a Knoxville neighborhood. Concordantly with data collection, these programs and partnerships also present novel opportunities to educate the community about locally important mosquito-borne diseases. We hope that these findings encourage other institutions in regions with similar challenges to consider developing similar community engagement programs, that include reciprocal partnerships, but we also acknowledge that doing so required ample collaborative effort among organizers of our program and the participating educators along with many hours preparing and presenting training materials, uninterrupted communications with participants, and time spent collecting and managing data.

## Data Availability

The datasets used and analyzed during the current study are available from the corresponding author on reasonable request. Data are also available on our data website: https://megabitess-tga.hub.arcgis.com/.

## Abbreviations

LACV: La Crosse virus
KKB: Keep Knoxville Beautiful
UTK: University of Tennessee Knoxville
MEGA:BITESS: Medical Entomology and Geospatial Analyses: Bringing Innovation to Teacher Education and Surveillance Studies
VBD: Vector-borne disease
GLMM: Generalized Linear Mixed Effect Model
BACI: Before-After-Control-Impact Analysis
Std Dev: Standard Deviation
